# Supplementary material for: ROR2 regulates self-renewal and maintenance of hair follicle stem cells
Source: Nat Commun. 2022 Aug 1;13:4449. doi: 10.1038/s41467-022-32239-7 (PMC9343661; doi:10.1038/s41467-022-32239-7)
Supplement: Supplementary file 1 — Supplementary Information [file 41467_2022_32239_MOESM1_ESM.pdf]

Supplementary Information

ROR2 Regulates Stem Cell Self-Renewal and Maintenance of Hair Follicle Stem Cells

Anthony Veltri[1,#], Christopher M. R. Lang[1,#], Gaia Cangiotti[1], Chim Kei Chan[1] and Wen-Hui Lien[1,*]

[1] *de Duve Institute, Université catholique de Louvain, 1200 Brussels, Belgium*

[#] Authors contributed equally

* Corresponding author: Wen-Hui Lien, Email address: wen-hui.lien@uclouvain.be

Supplementary Figures

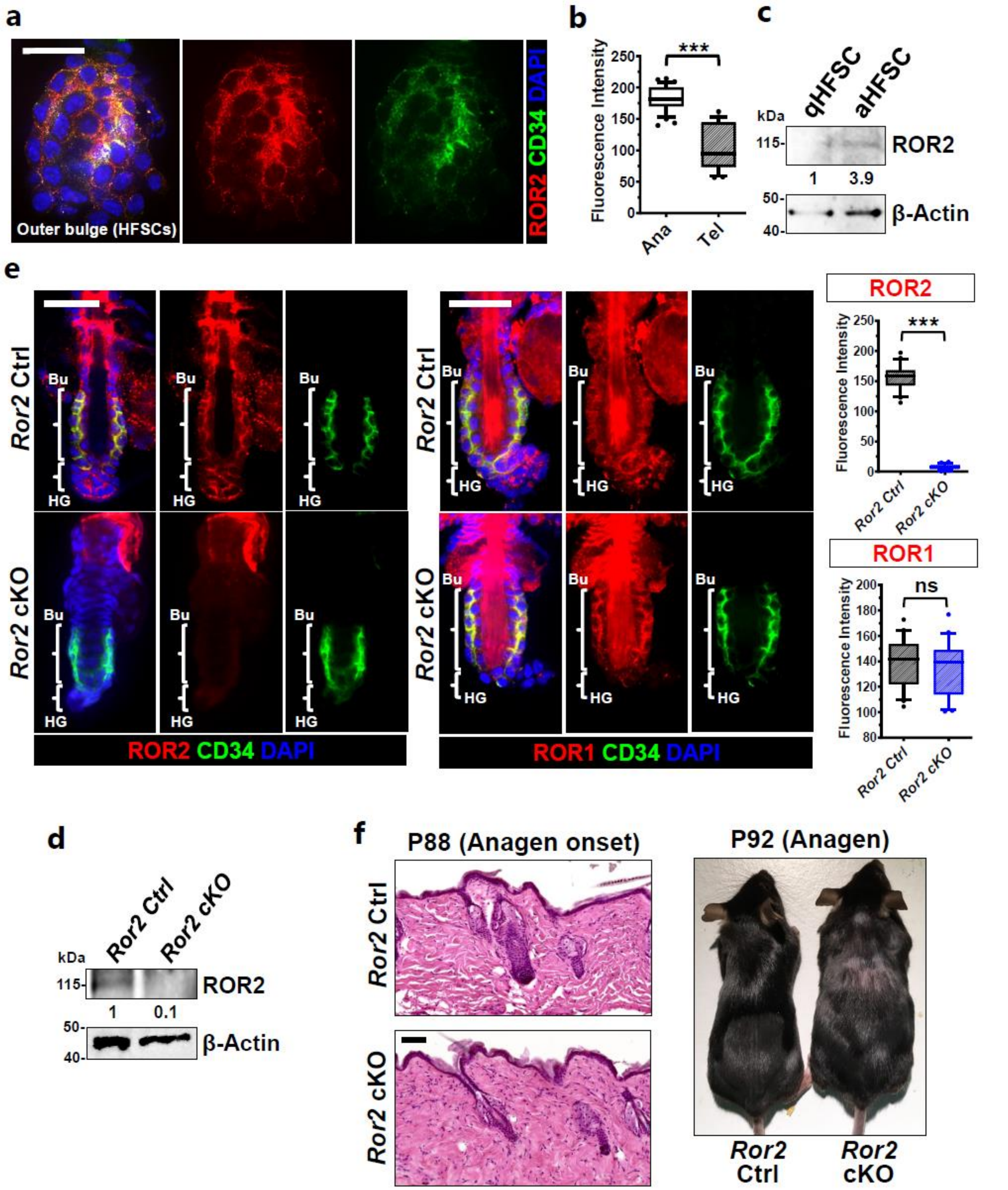

Supplementary Figure 1. *Ror2* depletion in HFSCs causes a delay of hair cycle entry. (**a**) Whole-mount immunofluorescence staining of mouse HFs for ROR2 and CD34. High magnification images confirm the expression of ROR2 at the cell membrane of CD34$^+$ HFSCs.

DAPI was used as a nuclear counterstain. (**b**) Quantification analysis of fluorescence intensity for ROR2 in the outer bulge of anagen and telogen HFs (as shown in Fig. 1a) demonstrates an increase of ROR2 expression in anagen HFSCs. (**c**) Immunoblotting analyses for ROR2 and β-Actin with the other set of FACS-purified quiescent (qHFSC) and activated (aHFSC) HFSCs. (**d**) Immunoblotting analyses of FACS-purified *Ror2* Ctrl and cKO HFSCs demonstrate the depletion of ROR2 in *Ror2* cKO HFSCs. (**e**) Whole-mount immunofluorescence staining of P32 *Ror2* Ctrl and cKO HFs for ROR2 or ROR1 together with CD34. Loss of ROR2 staining was detected in bulge and HG compartments of the *Ror2* cKO HF while ROR1 remained expressed. Quantification analyses of fluorescence intensity for ROR2 and ROR1 staining are shown at right. (**f**) *Ror2* cKO mice continuously display delayed anagen entry in the following hair cycle. (Left) H & E staining of *Ror2* Ctrl and cKO mouse back skins at the onset of the 2nd anagen (P88). (Right) The photograph of *Ror2* Ctrl and cKO mice at the 2nd anagen (P92). While *Ror2* Ctrl mouse recovered full hair coat, *Ror2* cKO mouse only show a half recovery. Scale bars in (a) represent 20 μm, and in (e) and (f) represent 50 μm. Data in (b) and (e) are reported as the median (the line within the box), the 25th to 75th percentiles (bottom and top lines of the box) and the 10th to 90th percentiles (bottom and top whiskers); n=33 (Ana) or 26 (Tel) regions over 9 (Ana) or 8 (Tel) independent HFs (b), n=21 (for ROR2) or 20 (for ROR1) regions over 7 independent HFs (e); ***p<0.0001; ns, not significant. Unpaired two-sided t-test. Data shown in (a), (c)-(f) are representative results from at least two independent experiments. Source data are provided as a Source Data file.

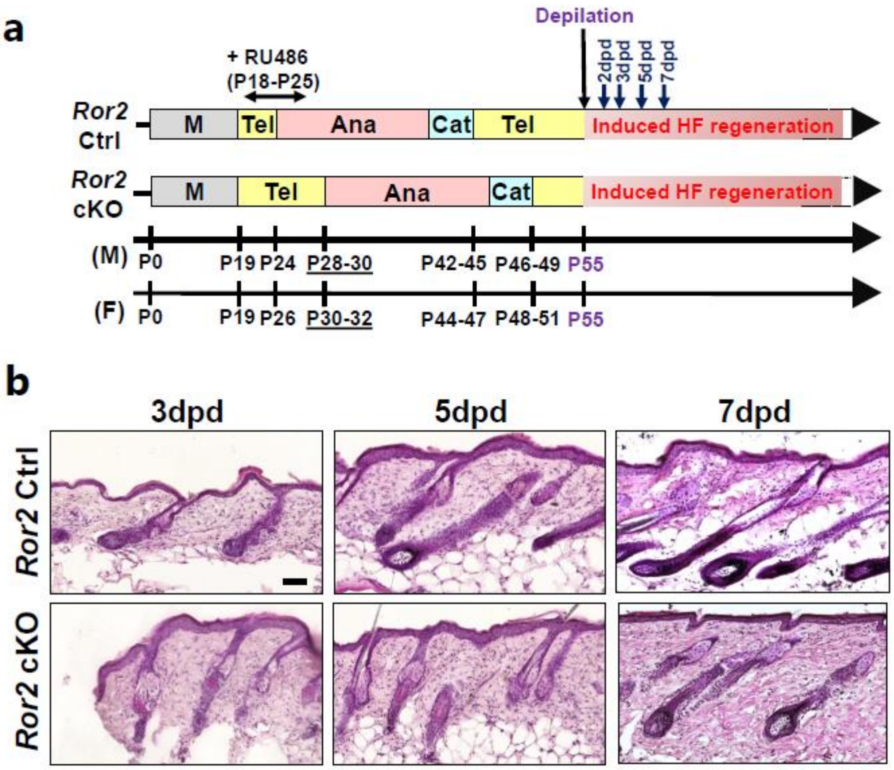

	3dpd		5dpd		7dpd	
	Ctrl	KO	Ctrl	KO	Ctrl	KO
Ana I/II	34	97	-	-	-	-
Ana III/IV	66	3	10	93	-	65
Ana V	-	-	90	7	12	35
Ana VI	-	-	-	-	88	-
Cat	-	-	-	-	-	-

Supplementary Figure 2. Deletion of *Ror2* in HFSCs results in a delay in depilation-induced anagen entry. (**a**) Schematic diagram illustrating application of depilation on back skins of *Ror2* Ctrl and cKO mice. Mice were treated with RU486 at P18-25, and depilation was performed on P55 mouse back skins during the 2nd telogen. Skins biopsies were collected 2 (24 h post-EdU labeling), 3, 5, and 7 days after depilation for analyses. Tel, telogen; Ana, anagen; Cat, catagen. (**b**) H&E staining of *Ror2* Ctrl and cKO HFs after 3, 5, and 7 days post-depilation (dpd) (top). Quantification of percentage of depilated HFs showing delayed anagen entry of *Ror2* cKO HFs (bottom). Data shown are representative results from at least two independent experiments. Scale bar represents 50 µm.

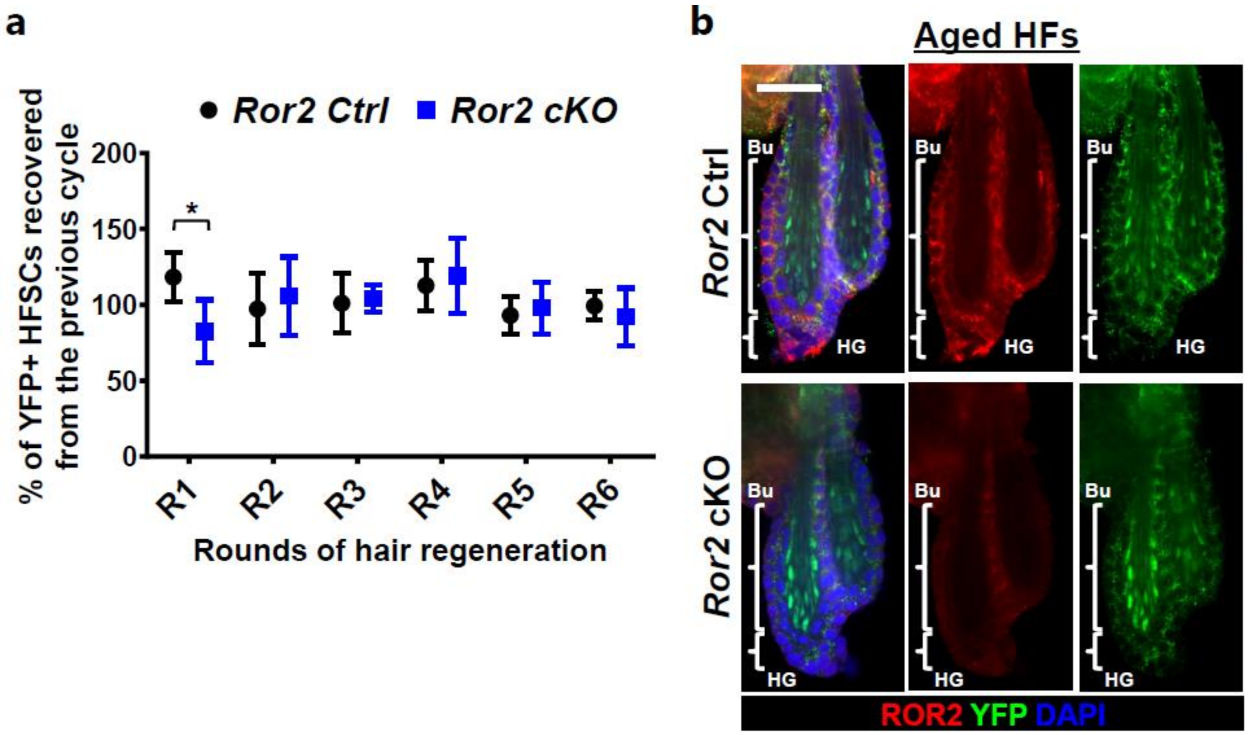

Supplementary Figure 3. Recovered *Ror2* cKO HFSCs are able to sustain the subsequent HF regeneration. (**a**) FACS analysis for YFP+ cells in integrin $\alpha6^{high}$/$CD34^{+}$ HFSC population from *Ror2* Ctrl and cKO HFs upon repetitive depilation. Dot plot shows percentages of YFP+ HFSCs recovered from the previous round (R) of depilation-induced HF regeneration. Except the 1st round of regeneration (R1), recovery rates of YFP+ *Ror2* cKO HFSCs from the previous HFSC pool are comparable with those of *Ror2* Ctrl HFSCs. Data are reported as mean ± SEM; *n*=4 biological independent animals; **p*=0.0372. Unpaired two-sided *t*-test. (**b**) Whole-mount immunofluorescence staining of *Ror2* Ctrl and cKO HFs at aged animals for ROR2 and YFP. Results are representative of at least two independent experiments. Note that YFP+ *Ror2* cKO HFSCs could be maintained in the HFs of aged animals. Scale bar represents 50 µm. Source data are provided as a Source Data file.

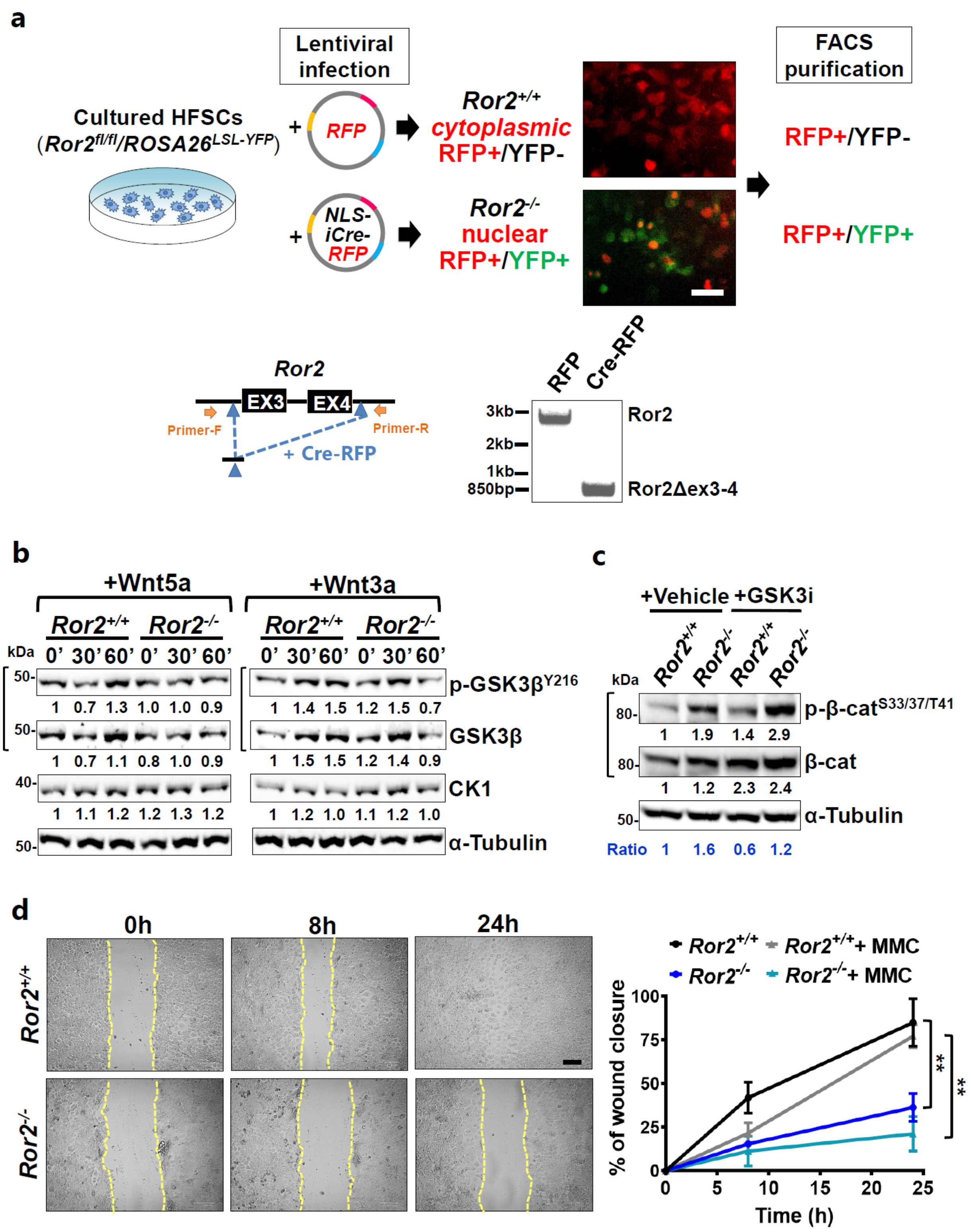

Supplementary Figure 4. Cultured HFSCs depleted for *Ror2* show impaired cell migration ability. (**a**) Schematic representation shows how *Ror2*$^{-/-}$ HFSCs were generated using the lentiviral infection strategy. HFSCs carrying *Ror2*$^{fl/fl}$ and *ROSA26*$^{LSL\text{-}YFP}$ were first established in culture and then infected by lentiviruses bearing RFP alone as a control or Cre-RFP

to transduce Cre recombinase. Control cells are RFP+/YFP- and referred as *Ror2*$^{+/+}$ HFSCs; Cre-transduced cells are RFP+/YFP+ and referred as *Ror2*$^{-/-}$ HFSCs. The depletion of *Ror2* in *Ror2*$^{-/-}$ HFSCs was confirmed by PCR with genomic DNA using primers recognizing external regions of *loxP* sites as indicated. (**b**) *Ror2*$^{+/+}$ and *Ror2*$^{-/-}$ HFSCs were starved overnight and then stimulated with 300 ng/ml of Wnt5a or 100 ng/ml of Wnt3a for 30 and 60 min before harvested for immunoblotting with antibodies recognizing indicated proteins. Values of fold change in the protein levels are shown under indicated bands. Data were normalized to α-Tubulin and calculated by comparing to *Ror2*$^{+/+}$ HFSCs at 0'. (**c**) Inhibition of GSK3β activity increased the total pool of β-catenin. Ratio of phosphorylated to total β-catenin is shown below in blue. (**d**) *Ror2*$^{-/-}$ HFSCs display impaired cell migration ability. Migration abilities of *Ror2*$^{+/+}$ and *Ror2*$^{-/-}$ HFSCs were examined by scratch wound migration assay. Photomicrographs of scratched wounds at the indicated time points. Yellow dashed lines denote the wound borders. Percentage of wound closure with or without mitomycin C (MMC) is shown at right. Data are reported as average ± s.d.; *n*=3 independent experiments; 24h: *Ror2*$^{+/+}$ *vs Ror2*$^{-/-}$, ***p*=0.0059; *Ror2*$^{+/+}$+MMC *vs Ror2*$^{-/-}$+MMC, ***p*=0.0012. Unpaired two-sided *t*-test. Scale bars in (a) and (d) represent 50 μm and 100 μm, respectively. Data in (b)-(d) shown are representative results from at least two independent experiments. Source data are provided as a Source Data file.

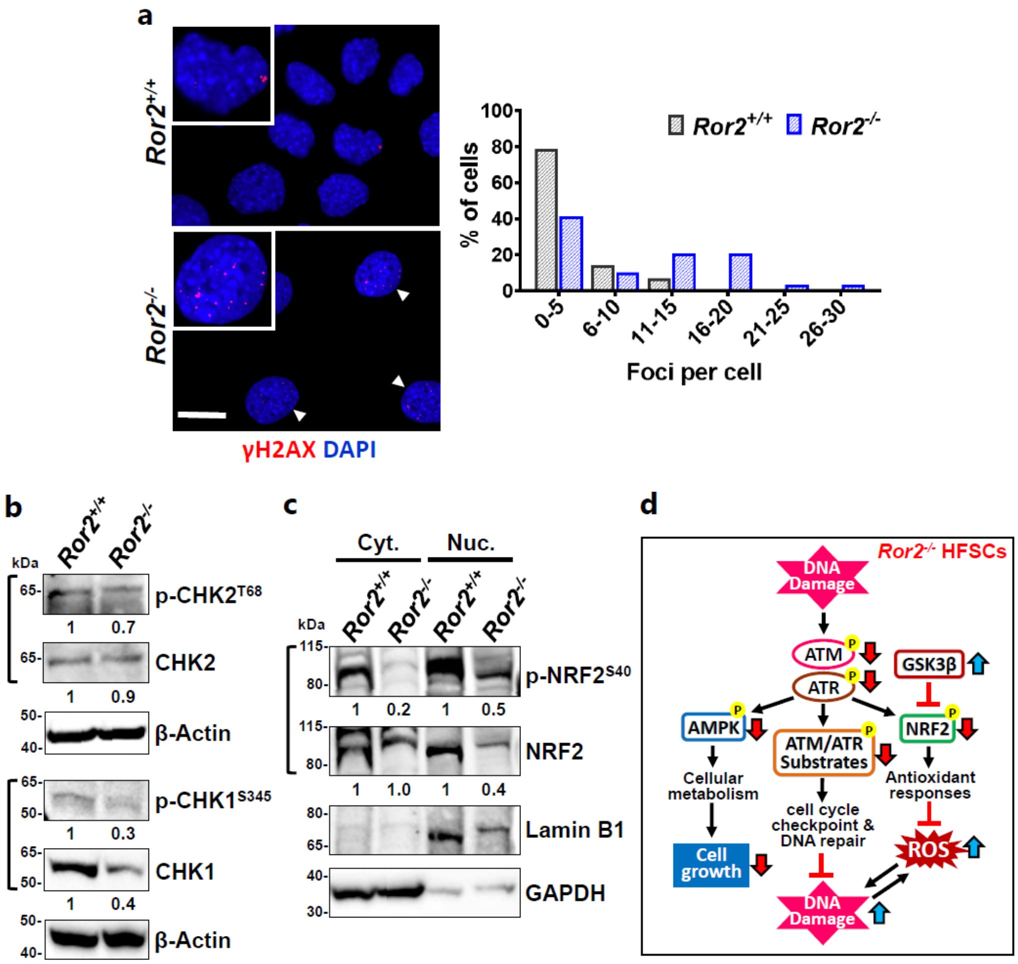

Supplementary Figure 5. *Ror2*$^{-/-}$ HFSCs show accumulation of DNA double-strand breaks and impairment in activation of ATM/ATR downstream effectors. (**a**) *Ror2*$^{-/-}$

HFSCs show an increase in DNA double-strand breaks. Immunofluorescence staining of *Ror2*$^{+/+}$ and *Ror2*$^{-/-}$ HFSCs for γH2AX. DAPI was used as a nuclear counterstain. Quantification of cells showing numbers of foci within the indicated ranges is shown at right. Arrowheads point to cells showing more over 6 foci. Scale bar represents 15 μm. (**b**) Downregulation of CHK2 and CHK1 in *Ror2*$^{-/-}$ HFSCs. Immunoblotting analyses of *Ror2*$^{+/+}$ and *Ror2*$^{-/-}$ HFSCs for activated and total proteins of CHK2 and CHK1. β-Actin was used as loading control. (**c**) Nuclear translocation of activated NRF2 was reduced in *Ror2*$^{-/-}$ HFSCs. Immunoblotting analyses of cytoplasmic (Cyt.) and nuclear (Nuc.) fraction of *Ror2*$^{+/+}$ and *Ror2*$^{-/-}$ HFSCs for activated and total NRF2 proteins. Lamin B and GAPDH were used as internal controls for nuclear and cytoplasmic fraction, respectively. (**d**) Schematic diagram summarizing the effects of *Ror2* depletion in HFSCs. In response to DNA damage, without the presence of ROR2, ATM and ATR fail to get activated, which in turn causes the impairment in the activation of their downstream substrates and effectors, eventually leading to slow cell growth, accumulation of unrepaired DNA damage, and ROS elevation. Data shown in (a)-(c) are representative results from at least two independent experiments. Source data are provided as a Source Data file.

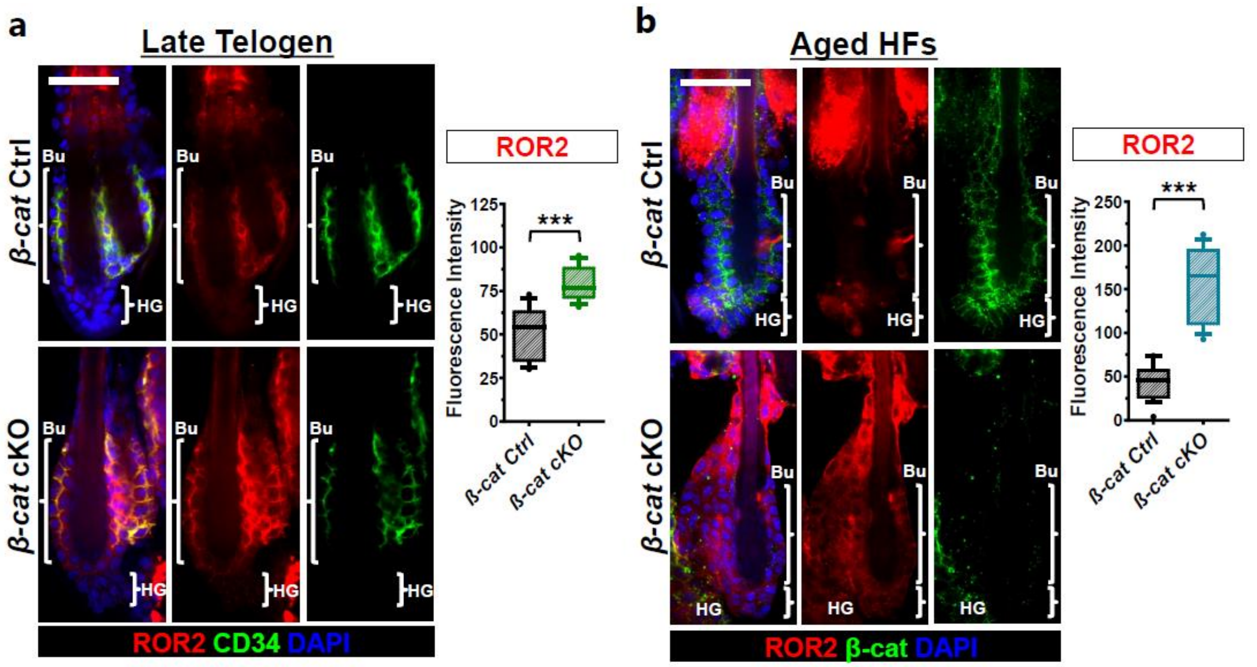

Supplementary Figure 6. ROR2 expression are elevated in *β-cat* cKO HFSCs. (**a-b**) Whole-mount immunofluorescence staining of *β-cat* Ctrl and cKO HFs at late telogen for ROR2 and CD34 (a) or at aged animals for ROR2 and β-catenin (b). Quantification analyses of fluorescence intensity for ROR2 staining are shown at right. Data are reported as the median (the line within the box), the 25th to 75th percentiles (bottom and top lines of the box) and the 10th to 90th percentiles (bottom and top whiskers); *n*=15 (Late Tel) or 18 (Aged) regions over 5 (Late Tel) or 6 (Aged) independent HFs; ****p*<0.0001. Unpaired two-sided *t*-test. Data shown are representative results from at least two independent experiments. Source data are provided as a Source Data file. Scale bars represent 50 μm.

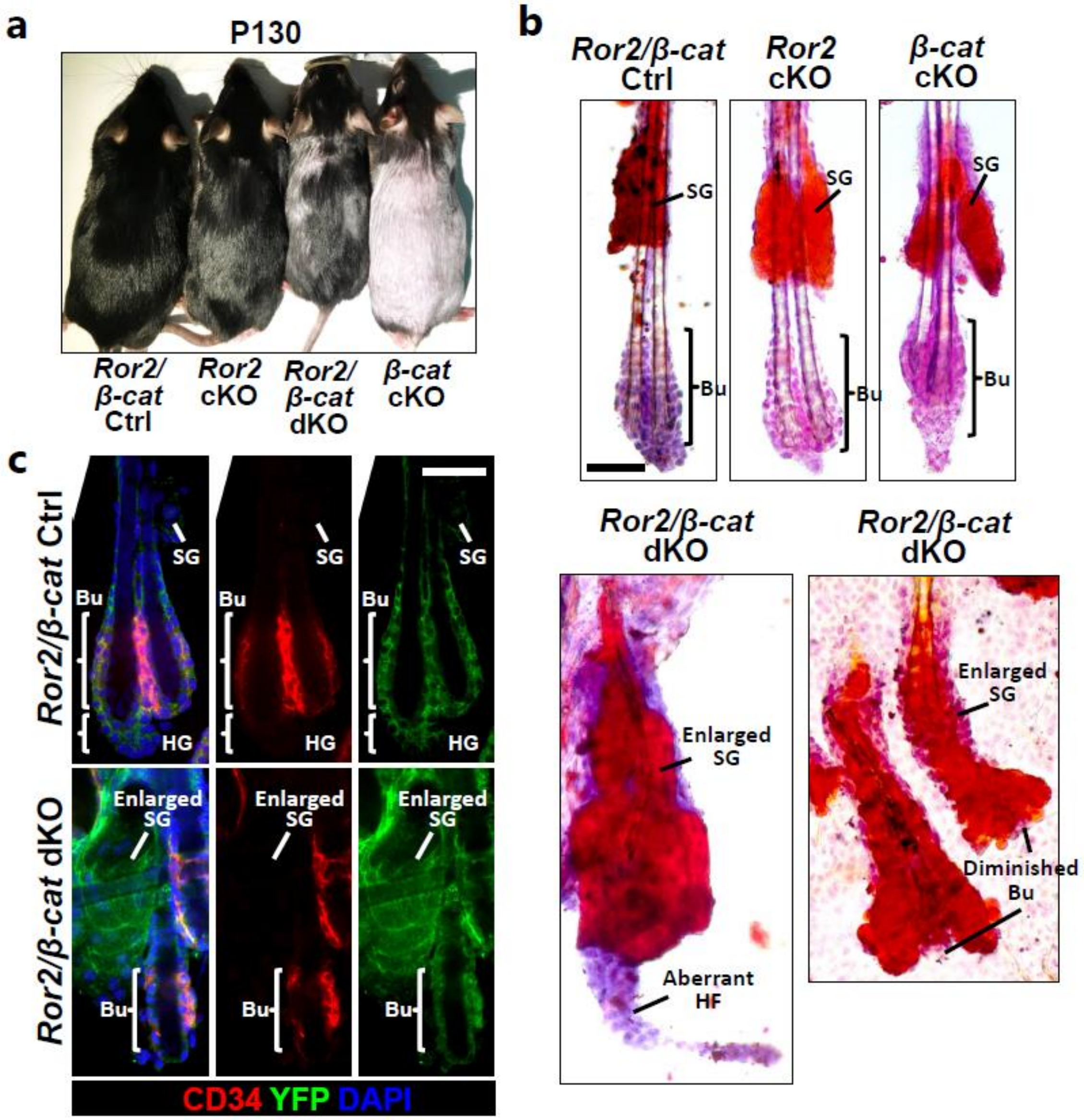

Supplementary Figure 7. *Ror2* depletion in *β-cat* cKO HFSCs causes *β-catenin*-null HFSCs differentiating towards sebocyte fate. (**a**) The photography of *Ror2/β-cat* Ctrl, *Ror2* cKO, *β-cat* cKO and *Ror2/β-cat* dKO mice at P130 is shown. (**b**) Oil Red O staining of sebocytes on whole-mount skins from an additional set of *Ror2/β-cat* Ctrl, *Ror2* cKO, *β-cat* cKO and *Ror2/β-cat* dKO mice. *Ror2/β-cat* dKO HFs show enlarged sebaceous glands (SGs) accompanied with aberrant hair follicles (HFs) or diminished bulge (Bu) as shown. (**c**) Whole-mount immunofluorescence staining of *Ror2/β-cat* Ctrl and dKO HFs for CD34 and YFP. Lineage tracing with YFP showed that enlarged SGs in *Ror2/β-cat* dKO HFs were YFP+ and accompanied with diminished bulge compartments. Scale bars in (b) and (c) represent 50 μm. Data shown in (b)-(c) are representative results from at least two independent experiments.

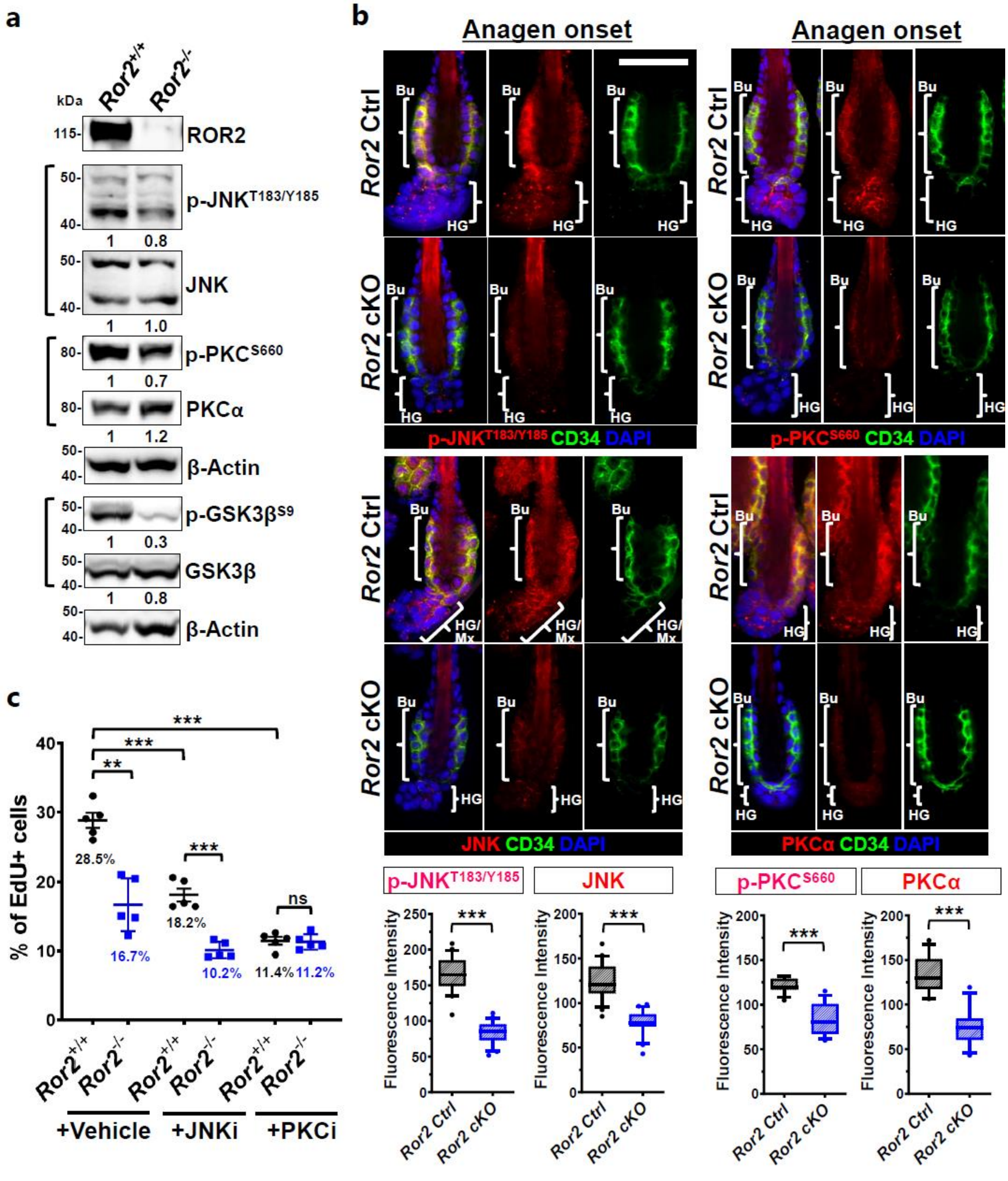

Supplementary Figure 8. The expression of JNK and PKC in HFSCs is ROR2-dependent. (**a**) ROR2 downstream effectors, JNK and PKC, were downregulated in cultured *Ror2*$^{-/-}$ HFSCs. Immunoblotting analyses for indicated proteins with *Ror2*$^{+/+}$ and *Ror2*$^{-/-}$ HFSCs that were cultured in the medium promoting proliferation. Uncropped images of blots are shown in Fig. S11. (**b**) Total and activated forms of JNK and PKC proteins were significantly reduced in *Ror2* cKO HFs at anagen onset. Whole-mount immunofluorescence staining of P32 *Ror2* Ctrl and cKO

HFs for JNK$^{T183/Y185}$, JNK, PKC^{S660}, or PKCα with CD34. Quantification analyses of fluorescence intensity for these proteins are shown at bottom. Data are reported as the median (the line within the box), the 25th to 75th percentiles (bottom and top lines of the box) and the 10th to 90th percentiles (bottom and top whiskers); n=21 (for p-JNK and JNK), 15 (for p-PKC) or 12 (for PKC) regions over 7 (for p-JNK and JNK), 5 (for p-PKC) or 4 (for PKC) independent HFs; ***p<0.0001. Scale bar represents 50 μm. (**c**) PKC inhibition erases the difference in cell proliferation between *Ror2*$^{+/+}$ and *Ror2*$^{-/-}$ HFSCs. *Ror2*$^{+/+}$ and *Ror2*$^{-/-}$ HFSCs were incubated with DMSO (vehicle), 2 μM of SP600125 (JNKi), or 0.5 μM of GF109203X (PKCi) for 24 h and then labeled with EdU for 4 h prior to examination. Note that the difference in cell proliferation between *Ror2*$^{+/+}$ and *Ror2*$^{-/-}$ HFSCs is erased by inhibition of PKC, but not JNK. Data are reported as mean ± SEM; n=5 independent experiments; **p=0.0007, ***p<0.0005; ns, not significant. Unpaired two-sided t-test. Data shown in (a)-(b) are representative results from at least three independent experiments. Source data are provided as a Source Data file.

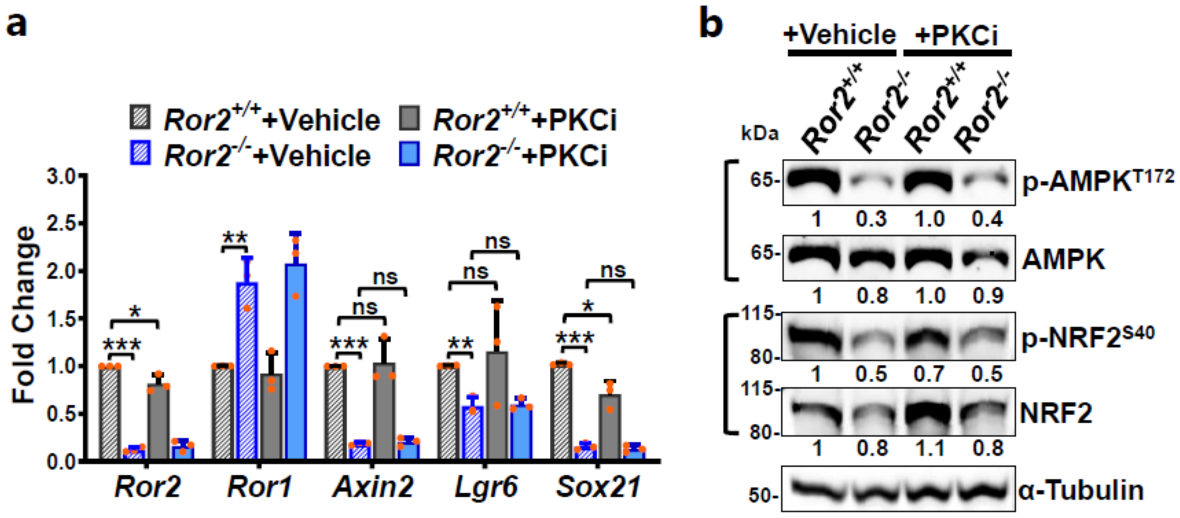

Supplementary Figure 9. Inhibition of PKC results in NRF2 inactivation, but shows very little effects on Wnt/β-catenin signaling and AMPK activation. (**a**) Inhibition of PKC activity did not influence the expression of canonical Wnt target genes in HFSCs. Real-time PCR analyses of *Ror2*$^{+/+}$ and *Ror2*$^{-/-}$ HFSCs treated with DMSO (vehicle) or 1 μM of GF109203X (PKCi) for 24 h for indicated genes. As shown, PKC inhibition showed no significant changes on the expression of Wnt target genes in both *Ror2*$^{+/+}$ and *Ror2*$^{-/-}$ HFSCs. Data are reported as average ± s.d.; n=3 independent experiments; *p<0.05, **p<0.005, ***p<0.0005; ns, not significant. Unpaired two-sided t-test. (**b**) Inhibition of PKC compromised NRF2 activity, but showed no impact on AMPK activation. Immunoblotting analyses for indicated proteins with *Ror2*$^{+/+}$ and *Ror2*$^{-/-}$ HFSCs that were treated with DMSO (vehicle) or 1 μM of GF109203X (PKCi) for 24 h. Results are representative of at least two independent experiments. Source data are provided as a Source Data file.

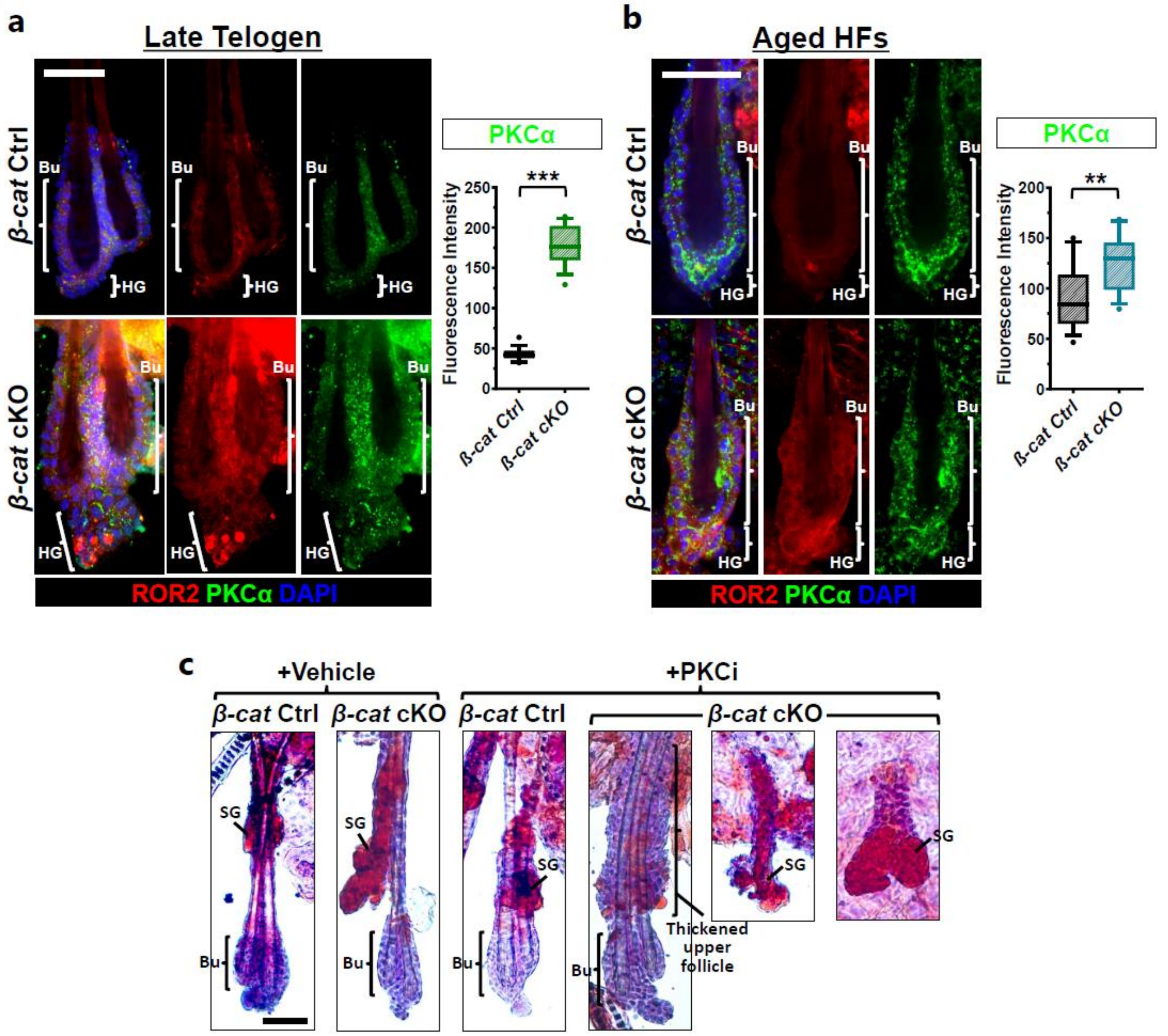

Supplementary Figure 10. PKC inhibition in *β-cat* cKO HFSCs leads to the loss of HFSCs and wrong fate differentiation. (**a-b**) PKC expression is increased in *β-cat* cKO HFSCs. Whole-mount immunofluorescence staining of *β-cat* Ctrl and cKO HFs at late telogen (a) or at aged animals (b) for ROR2 and PKCα. Quantification analyses of fluorescence intensity for PKCα staining are shown at right. Data are reported as the median (the line within the box), the 25th to 75th percentiles (bottom and top lines of the box) and the 10th to 90th percentiles (bottom and top whiskers); n=15 (Late Tel) or 18 (Aged) regions over 5 (Late Tel) or 6 (Aged) independent HFs; **p=0.0025; ***p<0.0001. Unpaired two-sided t-test. (**c**) Oil Red O staining of sebocytes on 3-week post-treated whole-mount *β-cat* Ctrl and cKO skins with acetone (vehicle) or GF109203X (PKCi). Note that only PKCi-treated *β-cat* cKO HFs show enlarged sebaceous glands companied with thickened upper follicle or diminished bulge. Scale bars in (a)-(c) represent 50 µm. Data shown are representative results from at least two independent experiments. Source data are provided as a Source Data file.

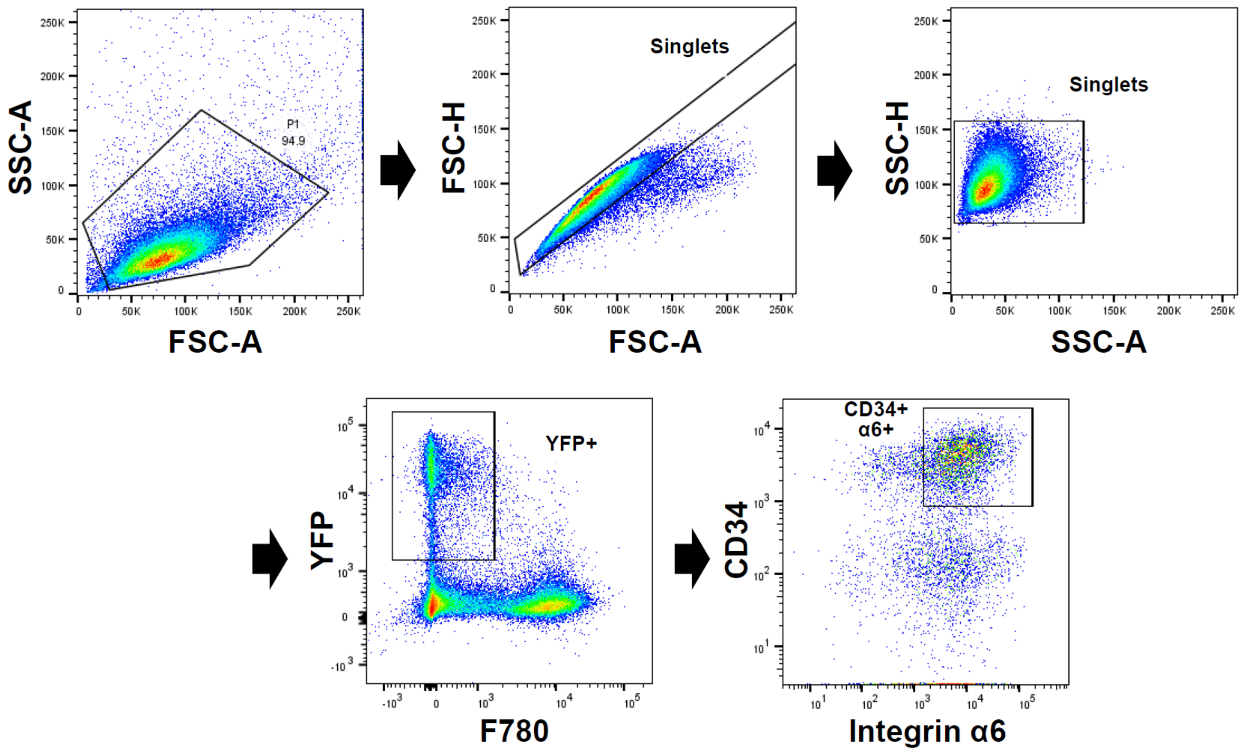

Supplementary Figure 11. FACS gating scheme for HFSC purification. FACS dot blots showing the sequential gating for YFP^{+}/$CD34^{+}$/integrin $α6^{high}$ HFSCs from mouse back skin.

Supplementary Tables

Supplementary Table 1: **Primers used for mRNA expression.**

Primer	Primer Sequence
Axin2-F	5'- ACT GAC CGA CGA TTC CAT GT -3'
Axin2-R	5'- CTG CGA TGC ATC TCT CTC TG -3'
Ccnd1-F	5'- CCC AAC AAC TTC CTC TCC TG -3'
Ccnd1-R	5'- TCC AGA AGG GCT TCA ATC TG -3'
Cd34-F	5'- AAG ACC ACA CCA GCC ATC TC -3'
Cd34-R	5'- GGG GAA GTC TGT GGT TGT GA -3'
Jun-F	5'- AGA ACT CGG ACC TTC TCA CG -3'
Jun-R	5'- TAG TGG TGA TGT GCC CAT TG -3'
Krt15-F	5'- GCT CGA GGG CCA GGA TGC TAA GAT -3'
Krt15-R	5'- TAC ACC GCT ACA CTC AGA AGG AAG -3'
Krt17-F	5'- AGG TTC ATC TCG GAC CTC CT -3'
Krt17-R	5'- CAT TGA GGT TCT GCA TGG TG -3'
Krt79-F	5'- GAG GAG AGC AGG ATG TCT GG -3'
Krt79-R	5'- CCA GAG AGA GGC CGT TCC -3'
Lgr5-F	5'- GCA AAC TTC CCA GAG CTC AA -3'
Lgr5-R	5'- GTT GCC GTC GTC TTT ATT CC -
Lgr6-F	5'- CTG ATG CAC CTG AAG CTC AA -3'
Lgr6-R	5'- ACA GCA CTG GTA GGC GTA GG -3'
Lhx2-F	5'- CGA CAC GGA GAC GAC CAT -3'
Lhx2-R	5'- TGC CAT TGC TTG TCT ACT GC -3'
NFATc1-F	5'- AAC GCC CTG ACC ACC GAT AGC ACT -3'
NFATc1-R	5'- CCC GGC TGC CTT CCG TCT CAT A -3'
Ppib2-F	5'- GTG AGC GCT TCC CAG ATG AGA -3'
Ppib2-R	5'- TGC CGG AGT CGA CAA TGA TG -3'
Ror1-F	5'- CAA ATG GCA AGA AAG TGG TG -3'
Ror1-R	5'- CGG CTG ACA GAA TCC ATC TT -3'
Ror2-F	5'- TCC AAG ACC TGG ACA CAA CA -3'
Ror2-R	5'- CCG AGC CGC ACA TAT AGA AC -3'
Rps16-F	5'- GAC GTC CCC TGG AGA TGA T -3'
Rps16-R	5'- CGA ATA TCC ACA CCA GCA AA -3'
Sox7-F	5'- CCC TTA CTC ACC GGA GTT CA -3'
Sox7-R	5'- GGG GAC ATC CAG AAA CAG AG -3'
Sox9-F	5'- CTC CGG CAT GAG TGA GGT -3'
Sox9-R	5'- TCG CTT CAG ATC AAC TTT GC -3'
Sox21-F	5'- CTG GGC AGC CTT ACT CTG ATT GT -3'
Sox21-R	5'- CTT TCC ACG CGT TCG TAA ACT G -3'
Tcf7-F	5'- GCC AGA AGC AAG GAG TTC AC -3'
Tcf7-R	5'- ACT GGG CCA GCT CAC AGT AT -3'
Tcf7l1-F	5'- TGG TCA ACG AAT CGG AGA AT -3'
Tcf7l1-R	5'- TCA CTT CGG CGA AAT AGT CG -3'
Tcf7l2-F	5'- CTC CAC AGC TCA AAG CAT CA -3'
Tcf7l2-R	5'- CAC CAC CTT CGC TCT CAT CT -3'
