# Supplementary material for: ROR2 regulates self-renewal and maintenance of hair follicle stem cells
Source: Nat Commun. 2022 Aug 1;13:4449. doi: 10.1038/s41467-022-32239-7 (PMC9343661; doi:10.1038/s41467-022-32239-7)
Supplement: Supplementary file 3 — Reporting Summary [file 41467_2022_32239_MOESM3_ESM.pdf]

nature research

Corresponding author(s): Wen-Hui Lien

Last updated by author(s): 6/27/2022

Reporting Summary

Nature Research wishes to improve the reproducibility of the work that we publish. This form provides structure for consistency and transparency in reporting. For further information on Nature Research policies, see Authors & Referees and the Editorial Policy Checklist.

Statistics

For all statistical analyses, confirm that the following items are present in the figure legend, table legend, main text, or Methods section.

n/a	Confirmed	
☐	☒	The exact sample size (*n*) for each experimental group/condition, given as a discrete number and unit of measurement
☐	☒	A statement on whether measurements were taken from distinct samples or whether the same sample was measured repeatedly
☐	☒	The statistical test(s) used AND whether they are one- or two-sided<br>*Only common tests should be described solely by name; describe more complex techniques in the Methods section.*
☒	☐	A description of all covariates tested
☐	☒	A description of any assumptions or corrections, such as tests of normality and adjustment for multiple comparisons
☐	☒	A full description of the statistical parameters including central tendency (e.g. means) or other basic estimates (e.g. regression coefficient) AND variation (e.g. standard deviation) or associated estimates of uncertainty (e.g. confidence intervals)
☐	☒	For null hypothesis testing, the test statistic (e.g. *F*, *t*, *r*) with confidence intervals, effect sizes, degrees of freedom and *P* value noted<br>*Give P values as exact values whenever suitable.*
☒	☐	For Bayesian analysis, information on the choice of priors and Markov chain Monte Carlo settings
☒	☐	For hierarchical and complex designs, identification of the appropriate level for tests and full reporting of outcomes
☒	☐	Estimates of effect sizes (e.g. Cohen's *d*, Pearson's *r*), indicating how they were calculated

Our web collection on statistics for biologists contains articles on many of the points above.

Software and code

Policy information about availability of computer code

Data collection	-Flow cytometry data were collected using FACSDiva v8.0.1 (BD Biosciences) on a FACSAria or LSR Fortesa flow cytometer (BD Biosciences)<br>-Microscopy images were acquired using Zen software v3.0 (Zeiss) on a Axio Vert.A1 fluorescence microscope (Zeiss) or Zen software v2.6 (Zeiss) on a Axio observer Z1 confocal microscope (Zeiss)<br>-Real-time PCR data were collected using CFX manager v3.1 software (Bio-Rad) on a Thermoblock 96 Silver Block Real-Time PCR system (Bio-Rad)<br>-Images of western blotting data were acquired using usion-Capt Adnance Solo 4S v16.16b software (Vilber) on a Fusion SL imaging system (Vilber)
Data analysis	-Flow cytometry data were analyzed by FlowJo v10.7.2<br>-Microscopy images were analyzed by Zen v3.0 (Zeiss) or ImageJ<br>-Western blotting signals were quantified by Image J or Fusion-Capt Adnance Solo 4S v16.16b software (Vilber)<br>-Graphs were presented using GraphPad Prism 8

For manuscripts utilizing custom algorithms or software that are central to the research but not yet described in published literature, software must be made available to editors/reviewers. We strongly encourage code deposition in a community repository (e.g. GitHub). See the Nature Research guidelines for submitting code & software for further information.

Data

Policy information about availability of data

All manuscripts must include a data availability statement. This statement should provide the following information, where applicable:

- Accession codes, unique identifiers, or web links for publicly available datasets
- A list of figures that have associated raw data
- A description of any restrictions on data availability

All data supporting the findings of this study are available in Source Data file.

Field-specific reporting

Please select the one below that is the best fit for your research. If you are not sure, read the appropriate sections before making your selection.

☒ Life sciences ☐ Behavioural & social sciences ☐ Ecological, evolutionary & environmental sciences

For a reference copy of the document with all sections, see nature.com/documents/nr-reporting-summary-flat.pdf

Life sciences study design

All studies must disclose on these points even when the disclosure is negative.

Sample size	The sample-size was determined based on the requirement to perform statistical tests and the availability of materials that were indicated in the figure legends. All quantitative data were collected from experiments performed in at least a triplicate.
Data exclusions	No samples were excluded from experiments.
Replication	All attempts for replication were successful. All measurements were performed at least 3 individual animals or independent experiments. The exact number of repeats are provided in each figure legends and in the Method and Material section.
Randomization	The mouse genotype determined the experimental group of each animal, so no randomization was used.
Blinding	No blinding were used in this study because the study is not investigating more than two populations.

Reporting for specific materials, systems and methods

We require information from authors about some types of materials, experimental systems and methods used in many studies. Here, indicate whether each material, system or method listed is relevant to your study. If you are not sure if a list item applies to your research, read the appropriate section before selecting a response.

Materials & experimental systems

n/a	Involved in the study	
☐	☒	Antibodies
☐	☒	Eukaryotic cell lines
☒	☐	Palaeontology
☐	☒	Animals and other organisms
☒	☐	Human research participants
☒	☐	Clinical data

Methods

n/a	Involved in the study	
☒	☐	ChIP-seq
☐	☒	Flow cytometry
☒	☐	MRI-based neuroimaging

Antibodies

Antibodies used	-Antibodies were used for FACS: Viability dye F780 (1:200, 65-0865), integrin α6 (1:500, PE-conjugated, clone GoH3, 12-0495), and CD34 (1:100, Alexa Fluor 660-conjugated, clone RAM34, 50-0341) from eBiosciences.<br>-Antibodies were used for immunoblotting:<br>ROR2 (Ror2) from Developmental Studies Hybridoma Bank; ROR1 (clone D6T8C, 16540), p-JNK-T183/Y185 (clone 81E11, 4668), JNK (clone 56G8, 9258), p-PKC-S660 (9371), PKCα (2056), Dvl2 (clone 30D2, 3224), p-LRP6-S1490 (2568), LRP6 (clone C5C7, 2560), p-GSK3β-S9 (9336), GSK3β (clone 27C10, 9315), p-β-catenin-S33/37/T41 (9561), Axin1 (clone C76H11, 2087), CK1 (2655), p-ATM-S1981 (clone D6H9, 5883), ATM (clone D2E2, 2873), p-ATR-S428 (2853), ATR (2790), p-CHK2-T68 (2661), CHK2 (2662), p-CHK1-S345 (clone 133D3, 2348), CHK1 (clone 2G1D5, 2360), p-AMPK-T172 (clone 40H9, 2535), AMPKα (2532), α-tubulin (2144), Lamin B1 (clone D9V6H, 13435), Vinculin (clone E1E9V, 13901) and p-ATM/ATR substrates (2851) from Cell Signaling Technology; p-NRF2-S40 (clone EP1809Y, ab76026) and NRF2 (ab137550) from Abcam; β-catenin (clone 15B8, C7207) and β-actin (clone AC-74, A2228) from Sigma-Aldrich; CD34 (Clone RAM34, 13-0341) from eBiosciences; Rac1 (clone 23A8, 05-389), Cdc42 (07-1466) and GAPDH (Clone 6C5, MAB374) from Merck-Millipore; Krt5 (905901) and Krt15 (833901) from Biolegend; p-GSK3β-Y216 (clone 13A, 612313) from BD Biosciences; HRP-conjugated secondary antibodies (715-035-150, 711-035-152, 712-035-150)

from Jackson ImmunoResearch. All primary antibodies used for immunoblotting were at 1:1000 dilution, and secondary antibodies at 1:4000.
-Antibodies were used for immunostaining:
ROR2 (1:100, Ror2) from Developmental Studies Hybridoma Bank; ROR1 (1:100, 16540), p-JNK-T183/Y185 (1:100, 4668), JNK (1:100, 9258), p-PKC-S660 (1:100, 9371), PKCα (1:100, 2056), γH2AX (1:200, BET A300-081A-M) from Cell Signaling Technology; GFP (1:1000, ab13970) from Abcam; β-catenin (1:100, C7207) from Sigma-Aldrich; CD34 (1:100, 13-0341) from eBiosciences; 8-oxoG (1:100, clone 483.15, MAB3560) from Merck-Millipore. Fluorescent dye-conjugated secondary antibodies (703-545-155, 715-545-150, 715-585-150, 711-545-152, 711-585-152, 712-545-150, 712-585-150) from Jackson ImmunoResearch.

Validation

Each selected antibodies has been quality-tested from vendor (details could be found in each indicated manufacturer's websites). In addition, all antibodies used for immunostaining and immunoblotting were compared with the information provided by indicated vendors and literatures to ensure the binding and localization of each antibodies with respect to their targets.

Eukaryotic cell lines

Policy information about cell lines

Cell line source(s)

293FT cell line (R70007) from Invitrogen (Thermo Fisher Scientific)

Authentication

Cell authentication test was not performed in this study

Mycoplasma contamination

Cell is tested negative for Mycoplasma

Commonly misidentified lines
(See ICLAC register)

No commonly misidentified cell lines were used in the study

Animals and other organisms

Policy information about studies involving animals; ARRIVE guidelines recommended for reporting animal research

Laboratory animals

All laboratory mice are housed in 12:12 light:dark light cycles at ambient temperature 20-24°C and humidity ranges of 45-65%. K15CrePGR and Ror2fl/fl mice on a C57BL/6J background were obtained from Jackson laboratory. Inducible knockout mouse lines were generated by crossing K15CrePGR79, Ror2fl/fl and/or Ctnnb1fl/fl80 , ROSA26LSL-YFP81. The strategy to generate Ror2 cKO and their control littermates was by breeding K15CrePGR+;Ror2+/fl;Rosa26LSL-YFP males with Ror2fl/fl; Rosa26LSL-YFP females. The control animals for Ror2 cKO mice were sex-matched Ror2 heterozygous (Cre+) littermates, or wildtype (Cre-) littermates only when the littermates of Ror2 cKO mice did not contain any Ror2 heterozygous mouse. Cre-recombinase activity was induced by intraperitoneal injection of RU486 (1 mg/mouse; TCI Europe N.V.) at P18, P21 and P24, and daily topical administration of 4% RU486 in ethanol from P21-P25. To induce synchronized HFSC activation, HF depilation was performed on anesthetized mice at P55. For PKC inhibitor treatment, 100 µg of GF109203X (R&D Systems) in acetone (1 µg/µl) or acetone alone was applied to P55 mouse back skin every other day for 3 days. Experiments were designed based on sex-, age- and strain-matched pairs, mainly littermates, and were repeated on ≥3 pairs of sample sets. Phenotype and obtained results were reproducible in both male and female pairs.

Wild animals

The study did not involve wild animals.

Field-collected samples

The study did not involve samples collected from the field.

Ethics oversight

Mice were housed and treated according to the guidelines of the University Animal Ethics Committee, Université Catholique de Louvain. All experimental procedures were conducted in compliance with animal welfare regulations of Belgium.

Note that full information on the approval of the study protocol must also be provided in the manuscript.

Flow Cytometry

Plots

Confirm that:

☒ The axis labels state the marker and fluorochrome used (e.g. CD4-FITC).

☒ The axis scales are clearly visible. Include numbers along axes only for bottom left plot of group (a 'group' is an analysis of identical markers).

☒ All plots are contour plots with outliers or pseudocolor plots.

☒ A numerical value for number of cells or percentage (with statistics) is provided.

Methodology

Sample preparation

Purification of hair follicle stem cells described previously (Lien et al., Nature Cell Biology, 2014)

Instrument

BD Aria2; BD LSRII

Software	BD FACSDiva v8.0.1; FlowJo v10.7.2
Cell population abundance	Sorting purity was checked on post-sorted population to confirm the cytometer setup. Abundance of the relative cells was >95%.
Gating strategy	Debris was gated out based on FSC-A vs SSC-A. Single cells were gated on FSC-A vs FSC-W, and then SSC-A vs SSC-W. Live cells were negative for Fixable Viability Dye eFluor 780. YFP+ cells were positive for Alexa F488. HFSCs were sorted on CD34+ and CD49f high. The FACS gating scheme is provided in Supplementary Figure 11.

☒ Tick this box to confirm that a figure exemplifying the gating strategy is provided in the Supplementary Information.
